# Non-surgical porto-mesenteric vein thrombosis is associated with worse long-term outcomes in inflammatory bowel diseases

**DOI:** 10.1093/gastro/gov012

**Published:** 2015-04-28

**Authors:** Zubin Arora, Xianrui Wu, Udayakumar Navaneethan, Bo Shen

**Affiliations:** ^1^Department of Internal Medicine, The Cleveland Clinic Foundation, Cleveland, OH, USA; ^2^Department of Gastroenterology and Hepatology, The Cleveland Clinic Foundation, Cleveland OH, USA

**Keywords:** inflammatory bowel diseases, portal vein thrombosis, outcomes, risk factors

## Abstract

**Objective:** Our aim was to assess the risk factors for non-surgery-related portal and mesenteric vein thrombosis (PMVT) and its impact on the outcomes of inflammatory bowel diseases (IBD).

**Methods:** All patients with a concurrent diagnosis of IBD and PMVT between January 2004 and October 2013 were identified from the electronic medical record (study group; *n = *20). Patients were matched for age, sex, and IBD phenotype with control IBD patients who had no PMVT, with a ratio of 1:3 (control group; *n = *60). Risk factors for PMVT and IBD-related outcomes at one year after diagnosis of PMVT were compared between the two groups.

**Results:** Of the 20 patients in the Study group, 6 (30%) had UC, 14 (70%) had CD and 11 (55%) were male. On multivariable analysis, inpatient status (odds ratio [OR] 6.88; 95% confidence interval [CI] 1.88–25.12) and baseline corticosteroid use (OR 4.39; 95% CI 1.27–15.19) were found to be independent risk factors for the development of PMVT. At one-year follow-up, PMVT patients were more likely to have an adverse outcome of IBD, including subsequent emergency room visit (26.3% *vs.* 1.7%; *P = *0.003), hospitalization for medical management (60.0% *vs.* 20.0%; *P = *0.001) or IBD-related surgery (65.0% *vs.* 26.7%; *P = *0.003) than the non-PMVT controls. In multivariable analysis, PMVT (OR 5.19; 95% CI 1.07–25.28) and inpatient status (OR 8.92; 95% CI 1.33–59.84) were found to be independent risk factors for poor outcome, whereas baseline immunomodulator use (OR 0.07; 95% CI 0.01–0.51) was found to be a protective factor.

**Conclusions:** IBD patients who were inpatients or receiving corticosteroid therapy had an increased risk of the development of PMVT. The presence of PMVT was associated with poor clinical outcomes in IBD.

## Background

Patients with inflammatory bowel diseases (IBD) are at an increased risk of developing venous thromboembolism (VTE) as a result of the hypercoagulable state induced by chronic bowel inflammation [1–5]. VTE presents most commonly with deep vein thrombosis (DVT) of the legs and pulmonary embolism [6]; however, other forms of VTE, such as portal or mesenteric vein thrombosis (PMVT), have also been described [7–11]. PMVT is frequently seen in IBD patients after intra-abdominal or pelvic surgery [12, 13].

Symptoms of PMVT are variable and patients may be asymptomatic with thrombosis discovered incidentally on abdominal imaging [11]. Symptoms are often non-specific and include fever, abdominal pain, nausea, and vomiting [12]. Rarely, patients can present with life-threatening complications, such as gastric and esophageal variceal bleeding as a result of portal hypertension [14, 15].

Previous studies have shown that the occurrence of PMVT in IBD patients in the post-operative setting was not associated with adverse clinical outcomes and most patients recovered uneventfully [12, 13, 16]; however, PMVT can also occur in IBD patients without any history of abdominal or pelvic surgery [14, 15, 17, 18]. The risk factors and the impact of non-surgery-related PMVT on the clinical course of IBD have not been systematically studied. The aim of this study was to evaluate the risk factors for non-surgery-related PMVT in IBD patients and the impact of this condition on the long-term outcomes of IBD.

## Methods

### Study subjects

After obtaining approval from the Institutional Review Board, records of all patients with a concurrent diagnosis of IBD and PMVT, who were regularly followed up in the IBD Center of the Cleveland Clinic Digestive Disease Institute between January 2004 and October 2013, were identified from the electronic medical record search using ICD-9 codes.

### Inclusion and exclusion criteria

Inpatients and outpatients with ulcerative colitis (UC) or Crohn’s disease (CD) who had one or more abdominal imaging indicative of PMVT were identified (*n = *20). Patients were matched for age, sex, and IBD phenotype with control IBD patients who had no PMVT on abdominal imaging and no previous history of PMVT in a consecutive manner from a cohort of IBD patients followed at our institution, with a ratio of 1:3 (*n = *60). Patients with cirrhosis, primary sclerosing cholangitis (PSC), malignancy or recent intra-abdominal or pelvic surgery for IBD- or non-IBD indications within 3 months prior to diagnosis of PMVT were excluded from the study.

### Diagnosis of PMVT

**Figure 1:**
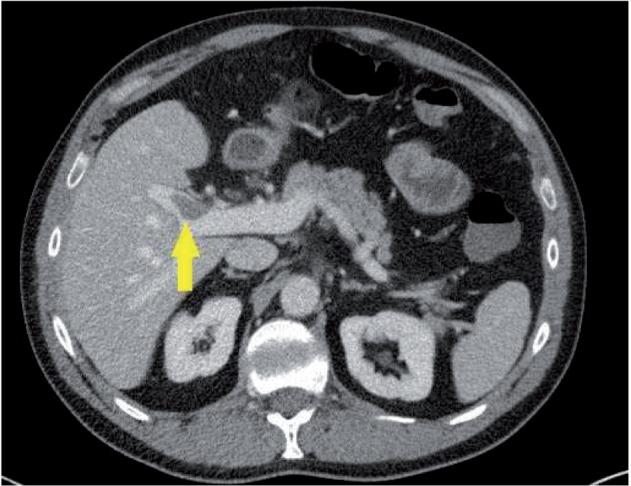
CT of the abdomen, demonstrating presence of thrombus in the portal vasculature.

The diagnosis of PMVT was made based on abdominal imaging in all patients. Imaging studies reviewed included Doppler ultrasound of the liver vasculature, abdominal computed tomography (CT) scan with contrast (Figure 1), and/or abdominal magnetic resonance imaging (MRI) with contrast. PMVT was defined as thrombosis involving either one or several of the following veins as seen on any abdominal imaging: portal vein and its branches, superior mesenteric vein, splenic vein, and inferior mesenteric vein. For the Control group, all abdominal imaging studies were negative for PMVT, including at least one abdominal CT scan or MRI with contrast.

### Demographic and clinical variables

Retrospective chart review was performed by one investigator (Z.A.) to extract relevant information about the patients, including demographics, clinical presentation, underlying risk factors, hypercoagulability work-up and anticoagulation therapy used for treatment of PMVT. IBD type (UC *vs.* CD) extent of disease (colonic, small bowel or both), IBD medications (5-aminosalicylic acid [5-ASA], corticosteroids, immunomodulators [azathioprine, 6-mercaptopurine, and methotrexate], anti-Tumor necrosis factor [TNF] biological therapy), chronic Non steroidal anti inflammatory drug (NSAID) use and the presence of any extra-intestinal manifestations (excluding PSC) were recorded. For CD patients, the disease behavior (stricturing or fistulizing) and perianal involvement was also characterized. In patients who had an upper endoscopy performed after diagnosis of PMVT, the presence or absence of esophageal or gastric varices was reviewed.

IBD activity at the time of diagnosis of PMVT was assessed, based on patients’ symptoms and endoscopic findings. The last colonoscopy performed at our institution prior to diagnosis of PMVT was reviewed for this purpose. Endoscopic findings were scored according to the Simple Endoscopic Score for Crohn's disease (SES-CD), and in UC according to the Mayo endoscopic score [19, 20]. When endoscopy results were unavailable, radiographic findings were used instead, based on the last CT enterography on record at our institution prior to diagnosis of PMVT. IBD was defined as being in remission if the patient did not have any pertinent gastrointestinal symptoms (abdominal pain, vomiting, diarrhea, or hematochezia) along with the absence of disease on endoscopy (SES-CD of 0–2 or Mayo endoscopic score of 0–1, as applicable) or radiography (absence of bowel wall thickening, mesenteric edema, or extensive lymphadenopathy).

### Outcomes

The primary outcomes of the study included subsequent IBD-related emergency room visits, hospitalizations or surgery at 1 year following diagnosis of PMVT. Poor outcome was defined as occurrence of any of the above events either alone or in combination with others. The need for corticosteroids and escalation of medical therapy for IBD in the year following the diagnosis of PMVT were also compared. Escalation of medical therapy was defined as either an increase in dose of ongoing medications, or the addition of immunomodulator/biological agent to anti-inflammatory therapy or the addition of anti-TNF biological therapy to immunomodulator therapy [21]. The secondary outcome was the risk factors associated with the development of PMVT.

### Statistical analysis

Descriptive statistics were computed for all variables. These included means and standard deviations or medians and interquartile ranges (IQR) for continuous factors, and frequencies for categorical factors. Comparisons between the two groups were made by using the 2-tailed *t*-test or Wilcoxon rank sum test, as appropriate, for continuous variables and chi-squared test or Fisher's exact test, as appropriate, for categorical variables. Multivariate logistic regression analyses were performed to evaluate factors associated with the development of PMVT as well as the risk for the poor IBD outcome. All statistical analyses were performed using SPSS software version 16 (SPSS, Chicago, IL, USA). A *P-*value lower than 0.05 was considered statistically significant.

## Results

A total of 80 patients were included in the investigation: 20 in the Study group (IBD patients with non-surgery-related PMVT) and 60 in the Control group (IBD patients without PMVT). The median duration of follow-up was 29.5 months (IQR = 20–45.7).

### Comparison between the study and control groups

Table 1 presents a summary of the demographic and clinical characteristics of the patients with and without PMVT. Of the 20 patients in the Study group, 6 (30%) had UC and 14 (70%) had CD and 11 (55%) were male. On univariable analysis, there were no significant differences in IBD duration, body mass index, tobacco use, oral contraceptives use, chronic NSAID use, extra-intestinal manifestations, or IBD-disease activity between the Study and Control groups

**Table 1.** Demographics and clinical characteristics

Characteristics	Study group (*n = *20)	Control group (*n = *60)	*P-*value
Male, *n* (%)	11 (55.0%)	33 (55.0%)	1.0
Age at diagnosis of PMVT, years	44.9 ± 17.0	45.5 ± 18.0	0.89
Duration of IBD, years	7.9 (1.6–29.4)	10.8 (5.5–20.8)	0.3
Underlying IBD, *n* (%)			1.0
UC	6 (30.0%)	18 (30.0%)	
CD	14 (70.0%)	42 (70.0%)	
IBD activity, *n* (%)			0.15
Remission	5 (25.0%)	26 (43.3%)	
Active	15 (75.0%)	34 (56.7%)	
UC extensive colitis, *n* (%)	6 (30.0%)	17 (28.3%)	0.89
CD with colon involvement, *n* (%)	11 (55.0%)	34 (56.7%)	0.9
Stricturing or fistulizing CD, *n* (%)	12 (60.0%)	33 (55.0%)	0.7
Extra-intestinal manifestations, *n* (%)	0 (0%)	8 (13.6%)	0.2
Baseline 5-ASA use, *n* (%)	8 (40.0%)	26 (43.3%)	0.8
Baseline corticosteroid use, *n* (%)	13 (65.0%)	13 (21.7%)	**<0.001**
Baseline immunomodulator use, *n* (%)	3 (15.0%)	14 (23.3%)	0.54
Baseline biologics, *n* (%)	5 (25.0%)	12 (20.0%)	0.75
Inpatient status at presentation, *n* (%)	12 (60.0%)	7 (11.7%)	**<0.001**
Chronic NSAID use, *n* (%)	9 (45.0%)	18 (30.0%)	0.28
Tobacco use, *n* (%)	10 (50.0%)	27 (45.0%)	0.7
Past history of DVT, *n* (%)	1 (5.0%)	4 (6.7%)	1.0
Body mass index, kg/m^2^	24.9 ± 6.8	25.0 ± 5.5	0.99
Oral contraceptive pills, *n* (%)	3 (15.0%)	4 (6.7%)	0.36
Hormone replacement therapy, *n* (%)	0 (0%)	2 (3.3%)	1.0

Continuous values presented as mean ± standard deviation or medians (interquartile ranges). Bold font indicates statistical significance for *P*-values *<* 0.05.

5-ASA = 5-aminosalicylic acid; CD = Crohn’s disease; DVT: deep vein thrombosis; IBD = inflammatory bowel disease; NSAID = non-steroidal anti-inflammatory drugs; PMVT = porto-mesenteric vein thrombosis; UC = ulcerative colitis.

### Risk factors for PMVT

On univariable analysis, patients in the Study group were more likely to be on corticosteroids at baseline (65.0% *vs.* 21.7%; *P < *0.001) and were more likely to be admitted to the hospital on presentatio*n* (60.0% *vs.* 11.7%; *P < *0.001) than patients in the Control group. On multivariable analysis, inpatient status and baseline corticosteroid use were found to be independent risk factors for development of PMVT (Table 2).

**Table 2.** Multivariable analysis: risk factors associated with PMVT development

Variables	Odds ratio	95% CI	*P-*value
Inpatient status at presentation	6.88	1.88–25.12	0.004
Baseline corticosteroid use	4.39	1.27–15.19	0.02
IBD activity (active *vs.* remission)	1.74	0.46–6.54	0.41

CI = confidence interval; IBD = inflammatory bowel disease; PMVT = porto-mesenteric vein thrombosis.

Among the 12 patients who were diagnosed with PMVT as inpatients, interruption of pharmacological DVT prophylaxis was observed in 3 (25%). Hypercoagulability work-up was available in 11 patients in the Study group, of which 5 (45.4%) tested positive. More specifically, 3 patients tested positive for lupus anticoagulant, 1 for heterozygous factor V Leiden, and 1 had decreased protein C levels.

### Risk factors for poor clinical outcomes

Table 3 summarizes the differences in the outcomes of patients with and without PMVT. At 1-year follow-up after diagnosis, PMVT patients were significantly more likely to subsequently require corticosteroids (47.4% *vs.* 23.3%; *P = *0.04), have an IBD-related emergency room visits (26.3% *vs.* 1.7%; *P = *0.003), require hospitalization for medical management (60.0% *vs.* 20.0%; *P = *0.001) or undergo IBD-related surgery (65.0% *vs.* 26.7%; *P = *0.003) than the non-PMVT controls.

**Table 3.** IBD outcomes one year after diagnosis: PMVT *vs.* non-PMVT patients

Outcomes	Study group(*n = *20)	Control group (*n = *60)	*P-*value
Escalation of medical therapy, *n* (%)	3 (15.0%)	11 (18.3%)	1.0
Subsequent corticosteroid use, *n* (%)	9 (45.0%)	14 (23.3%)	**0.044**
IBD-related emergency room visit, *n* (%)	5 (25.0%)	1 (1.7%)	**0.003**
IBD-related hospitalization, *n* (%)	12 (60.0%)	12 (20.0%)	**0.001**
IBD-related surgery, *n* (%)	13 (65.0%)	16 (26.7%)	**0.003**

Bold font indicates statistical significance for *P*-values *<* 0.05.

IBD = inflammatory bowel disease; PMVT = porto-mesenteric vein thrombosis.

Univariable analysis of the risk factors associated with poor IBD outcomes was performed (Table 4). Patients with poor outcomes were significantly younger and had a shorter duration of IBD than those with good outcomes. The presence of PMVT, baseline corticosteroid therapy and inpatient status at presentation were associated with poor IBD outcomes, whereas immunomodulator use at baseline was associated with good outcomes. Among patients with PMVT, there was no statistical difference in the rate of poor outcomes between patients who received anticoagulation and those who did not (92.3% *vs.* 71.4%; *P = *0.27).

**Table 4.** Univariable analysis: risk factors associated with 1-year poor outcomes

Characteristics	Goodoutcomes (*n = *42)	Poor outcomes (*n =* 38)	*P-*value
Male, *n* (%)	26 (61.9%)	18 (47.4%)	0.19
Age at time of abdominal imaging, years	49.6 ± 17.8	40.6 ± 16.3	**0.021**
PMVT patients, *n* (%)	3 (7.1%)	17 (44.7%)	**<0.001**
Duration of IBD, years	12.6 (6.9–25.5)	7.7 (1.6–19.1)	**0.011**
Underlying IBD, *n* (%)			0.43
UC	11 (26.2%)	13 (34.2%)	
CD	31 (73.8%)	25 (65.8%)	
IBD activity, *n* (%)			0.087
Remission	20 (47.6%)	11 (28.9%)	
Active	22 (52.4%)	27 (71.1%)	
UC extensive colitis, *n* (%)	11 (26.2%)	12 (31.6%)	0.6
CD with colon involvement, *n* (%)	25 (59.5%)	20 (52.6%)	0.54
Stricturing or fistulizing CD, *n* (%)	24 (57.1%)	21 (55.3%)	0.87
Extra-intestinal manifestations, *n* (%)^a^	5 (11.9%)	3 (7.9%)	0.73
Baseline 5-ASA use, *n* (%)	17 (40.5%)	17 (44.7%)	0.7
Baseline corticosteroid use, *n* (%)	8 (19.0%)	18 (47.4%)	**0.007**
Baseline immunomodulator use, *n* (%)	13 (31.0%)	4 (10.5%)	**0.026**
Baseline biologics, *n* (%)	9 (21.4%)	8 (21.1%)	0.97
Inpatient status on presentation	2 (4.8%)	17 (44.7%)	**<0.001**
Chronic NSAID use, *n* (%)	12 (28.6%)	15 (39.5%)	0.35
Tobacco use, *n* (%)	21 (50.0%)	16 (42.1%)	0.48
History of DVT, *n* (%)	3 (7.1%)	2 (5.3%)	1.0
Body mass index, kg/m^2^	25.9 ± 4.6	23.9 ± 6.8	0.13
Oral contraceptive pills, *n* (%)	2 (4.8%)	5 (13.2%)	0.25
Hormone replacement therapy, *n* (%)	2 (4.8%)	0 (0%)	0.5

Continuous values presented as mean ± standard deviation or medians (interquartile ranges). Bold font indicates statistical significance for *P*-values *<* 0.05.

^a^Excluding primary sclerosing cholangitis.

5-ASA = 5-aminosalicylic acid; CD = Crohn’s disease; DVT = deep vein thrombosis; IBD = inflammatory bowel disease; PMVT = porto-mesenteric vein thrombosis; NSAID = non-steroidal anti-inflammatory drugs; UC = ulcerative colitis.

On multivariable analysis, the presence of PMVT (odds ratio [OR] 5.19; 95% confidence interval [CI] 1.07–25.28) and inpatient status (OR 8.92; 95% CI 1.33–59.84) at presentation were found to be independent risk factors for poor outcomes, whereas the baseline use of immunomodulator (OR 0.07; 95% CI 0.01–0.51) was found to be a protective factor (Table 5).

**Table 5.** Multivariable analysis: Risk factors associated with 1-year poor outcomes

Characteristic	Odds ratio	95% confidence interval	*P-*value
Presence of PMVT	5.19	1.07–25.28	**0.041**
Age at PMVT diagnosis, every 5-year increase	0.78	0.65–0.95	**0.012**
Inpatient status at presentation	8.92	1.33–59.84	**0.024**
Baseline immunomodulator use	0.071	0.01–0.51	**0.009**

PMVT = porto-mesenteric vein thrombosis.

Of the 13 patients in the Study group who underwent IBD-related surgery in the 1-year follow-up period, 8 patients underwent small bowel resection, 1 had strictureplasty and 4 had subtotal or total colectomy. Out of the 16 patients in the Control group who underwent IBD-related surgery in the 1-year follow-up period, 5 underwent small bowel resection, 3 had strictureplasty, and 8 had partial or total colectomy.

### Treatment of PMVT

Of 20 patients in the Study group, 13 (65.0%) were treated with anticoagulation therapy. Warfarin was used in 10 patients (76.9%) and subcutaneous low molecular weight heparin was used in 3 (23.1%). The duration of anticoagulation was 6 months in 10 patients (76.9%), 12 months in 2 (15.4%), and lifelong therapy was initiated in 1 (7.7%). Upper endoscopy was performed in 9 patients (45%) and none of them was reported to have esophageal or gastric varices. Follow-up imaging was available in 16 patients and showed resolution of PMVT in 9 (56.3%) of them.

## Discussion

PMVT is a rare but potentially life-threating complication of IBD [14, 15]. Although seen more frequently after intra-abdominal or pelvic surgery, it can also be seen in patients outside the post-operative setting [8, 10, 18]. The present study describes the risk factors for development of PMVT in IBD patients outside the surgical setting and also attempts to determine its impact on their IBD outcomes. We found that inpatient status and corticosteroid therapy were risk factors for PMVT in IBD patients. Patients who developed PMVT had significantly worse clinical outcomes than those without PMVT, including IBD-related emergency room visits, hospitalization for medical management and need for IBD-related surgery. PMVT and inpatient status at presentation were found to be independently associated with worse outcomes.

The pathogenesis of PMVT appears to be multifactorial. Several local and systemic risk factors for PMVT have been described in the literature and include cirrhosis, intra-abdominal inflammatory conditions, malignancy and mechanical injury to the portal venous system [22]. It has been postulated that direct surgical trauma to the vasculature, superimposed on the background hypercoagulable state, may be the precipitating factor leading to thrombosis in the porto-mesenteric system in IBD patients undergoing intra-abdominal or pelvic surgery [13]. However, in non-surgical IBD patients, thrombus formation occurs without the additional stimulus of vascular trauma. We found that inpatient status and corticosteroid therapy were risk factors for the development of PMVT. This suggests that these patients may have more severe bowel inflammation, leading to activation of the coagulation pathways and subsequent thrombosis. Our results also suggest a possible protective effect of immunomodulator therapy. This may indicate a potential role of immune-mediated pathways in the pathogenesis of inflammation and coagulation, which needs to be studied further.

VTE in IBD patients has previously been shown to be associated with increased mortality [23]. However, in post-surgical IBD patients, previous studies have shown that development of PMVT is not associated with worse clinical outcomes and most patients recover without any long-term consequences [12, 13, 16]. In the present study, IBD patients who developed PMVT outside the surgical setting had significantly higher rates of subsequent IBD-related Emergency Room visits, hospitalizations and surgeries. This difference in outcomes between surgical and non-surgical PMVT patients provides further support for more severe inflammation in the non-surgical PMVT patients. Findings from studies in murine models of IBD have shown that inflammation and coagulation are closely inter-related, with one inducing and potentiating the other, thus leading to a vicious cycle [2, 24]. This vicious cycle of worsening inflammation in patients with non-surgery-related PMVT may potentially be the cause of worse outcomes; on the other hand, in patients with surgical PMVT, this vicious cycle is terminated by surgically removing the inflamed bowel from the body.

Our findings have several clinical implications. IBD patients who develop PMVT outside the surgical setting are at high risk of worse clinical outcomes; therefore, patients with risk factors for development of PMVT—especially hospitalized IBD patients and those on corticosteroid therapy—should be monitored closely. In these patients, a screening abdominal imaging technique, such as Doppler US, may be advocated. Previous studies have noted inadequate and sub-par DVT prophylaxis rates among hospitalized IBD patients, which increases the risk of thrombotic complications [25, 26]. In our study also, 25% patients with PMVT were noted as having interruptions in DVT prophylaxis. Although not evaluated in the present study, data from patients with cirrhosis and post-surgical IBD patients suggest that prophylactic subcutaneous unfractionated heparin or low molecular weight heparin may be effective in preventing PMVT and should be routinely administered in hospitalized IBD patients [12, 27]. Our findings add to the growing body of literature regarding the importance of providing adequate DVT prophylaxis to inpatients. In our study, hypercoagulability testing was abnormal in nearly 50% of patients for whom the test results were available. These results are in agreement with previous studies in which 25–50% of IBD patients who developed PMVT have been seen to have these abnormalities [8–11]. This is important, since the recommended duration of anticoagulation therapy may change and the risk for subsequent thromboembolic events may also be elevated in the presence of these abnormalities. Attempts should therefore be made to rule out an underlying inherited or acquired hypercoagulability disorder.

There are limitations to our study. There might have been a referral bias, as the study was conducted at a tertiary referral center. Due to this, patients in our study may have had more severe disease and may not be representative of those seen in the general community. Previous studies indicate that some patients with PMVT may be asymptomatic and therefore go undiagnosed [10, 11]. This leads to the possibility of selection bias, where only the symptomatic patients—presumably also with more severe IBD—were included in the study while the asymptomatic patients with less severe IBD went undiagnosed. Also, due to the retrospective nature of our study, hypercoagulability work-up and upper endoscopy results were not available for all patients; however, many of these issues are inherent to any retrospective study carried out at a tertiary center and could not be avoided. Finally, interaction effects might exist between the variables—such as between inpatient status and baseline corticosteroid use, included in the multivariate analysis—which posed a risk of overestimation of their predictive values; however, further efforts of assessing the interactions were not tried due to the exploratory nature of the analysis. To the best of our knowledge, this is the first study to demonstrate differences in IBD outcomes in patients with and without PMVT. We excluded patients with cirrhosis, PSC and malignancy in order to avoid possible confounding effects from these conditions. Also, the present study used objective clinical outcomes, which are less prone to bias.

In conclusion, we found that IBD patients who were receiving corticosteroid therapy or were admitted to the hospital had an increased risk of the development of PMVT. The presence of PMVT was associated with poor clinical outcomes in IBD. Patients with PMVT, therefore, warrant closer monitoring and follow-up.

## Funding

The research and education activity of Bo Shen, MD, is supported by the Ed and Joey Story Endowed Chair. The remaining authors declare no funding support.

*Conflict of interest statement*: none declared.
